# *Artemisia annua* Growing Wild in Romania—A Metabolite Profile Approach to Target a Drug Delivery System Based on Magnetite Nanoparticles

**DOI:** 10.3390/plants10112245

**Published:** 2021-10-21

**Authors:** Adina-Elena Segneanu, Catalin Nicolae Marin, Ioan Ovidiu-Florin Ghirlea, Catalin Vladut Ionut Feier, Cornelia Muntean, Ioan Grozescu

**Affiliations:** 1 Faculty of Physics, West University of Timisoara, Blvd. V. Parvan 4, 300223 Timisoara, Romania; catalin.marin@e-uvt.ro; 2 Faculty of Medicine, Victor Babes University of Medicine and Pharmacy Timisoara, 2 P-ta E. Murgu, 300041 Timisoara, Romania; ovidiu.ghirlea@gmail.com (I.O.-F.G.); catalinfeier10@gmail.com (C.V.I.F.); 3 Faculty of Industrial Chemistry and Environmental Engineering, Polytechnic University Timisoara, 2 P-ta Victoriei, 300006 Timisoara, Romania; cornelia.muntean@upt.ro (C.M.); ioangrozescu@gmail.com (I.G.)

**Keywords:** secondary metabolites, GC-MS, mass-spectra, bioactive compounds, nano-carrier system, magnetic nanoparticles

## Abstract

The metabolites profile of a plant is greatly influenced by geographical factors and the ecological environment. Various studies focused on artemisinin and its derivates for their antiparasitic and antitumoral effects. However, after the isolation and purification stage, their pharmaceutical potential is limited due to their low bioavailability, permeability and lifetime. The antibacterial activity of essential oils has been another topic of interest for many studies on this plant. Nevertheless, only a few studies investigate other metabolites in *Artemisia annua*. Considering that secondary metabolites act synergistically in a plant, the existence of other metabolites with antitumor and high immunomodulating activity is even more important. Novel nano-carrier systems obtained by loading herbs into magnetic nanoparticles ensures the increase in the antitumor effect, but also, overcoming the barriers related to permeability, localization. This study reported the first complete metabolic profile from wild grown Romanian *Artemisia annua*. A total of 103 metabolites were identified under mass spectra (MS) positive mode from 13 secondary metabolite categories: amino acids, terpenoids, steroids, coumarins, flavonoids, organic acids, fatty acids, phenolic acids, carbohydrates, glycosides, aldehydes, hydrocarbons, etc. In addition, the biological activity of each class of metabolites was discussed. We further developed a simple and inexpensive nano-carrier system with the intention to capitalize on the beneficial properties of both components. Evaluation of the nano-carrier system’s morpho-structural and magnetic properties was performed.

## 1. Introduction

Romanian phytotherapy has an ancient and very rich tradition based on a very wide diversity of medicinal plants. Thus, in the spontaneous flora of Romania there are about 800 species of medicinal plants. Additionally, plants of the genus Artemisia (*Asteraceae*) form part of this phytopharmacological treasure. 

*Artemisia annua* (common name: wormwood or năfurica in Romanian) is one of the ancient healing plants recognized in traditional medicine from Europe and Asia. Romanian traditional medicine has exploited its therapeutic properties: antihemorrhagic, antiseptic, antioxidant, digestive, antipyretic, immunomodulatory, antibacterial and antitumoral [1,2,3,4,5]. *Artemisia annua* is used also in Romania to prepare a digestive wine [1,2,3,4,5]. In traditional Asian pharmacopoeia, it was recommended, especially for the treatment of fevers and colds. 

Chemical screening of bioactive compounds isolated from this plant has highlighted the rich content in volatile compounds, terpenes, sesquiterpenes, alkaloids, flavonoids, coumarins and phenolic compounds. 

Studies on biological properties of secondary metabolites isolated from extracts of *Artemisia annua* have confirmed its therapeutic properties (antioxidant, antidiabetic, antiviral, antitumor, immunomodulatory, antiparasitic, antibacterial and antifungal activity) described in traditional medicine [4,5,6,7,8,9,10]. 

Additionally, the latest studies in the field aim to evaluate its potential application in the prevention and treatment of SARS-CoV 19 [6,7,8,9,10,11,12,13,14,15,16,17,18,19,20,21,22,23]. 

In recent years, numerous studies have been conducted on the main classes of phytoconstituents (phenolic compounds, alkaloids, terpenes and volatile compounds) in sweet wormwood. Special attention was paid to the isolation and chemical synthesis of artemisinin, considered the main bioactive compound of the plant, with antiparasitic activity. This was an important step in modern research in medicinal plants and in the fight against malaria, an infectious disease that affects the lives of millions of people [6,7,8,9,10,11,12,13,14,15,16,17,18,19,20]. According to World Health Organization estimates, malaria killed more than 400 million people globally in 2019 alone [24].

Artemisinin is widely used for malaria treatment, and recent studies demonstrated its antitumoral activity on several cancer cell lines. However, at present, the production costs of artemisinin are high. Moreover, its low bioavailability, permeability and life-time in biological media represent the main biomedical limitations [25].

It is known that the biological activity of a plant is due to the synergistic effect of different types of phytoconstituents [26,27]. Therefore, the chemical screening and complete identification of bioactive components from a plant is especially important. 

Recent studies have shown that the antitumor activity of the plant is not only due to artemisinin. This might be just one of the plant metabolites with high biological activity [6]. 

Although the plant has been studied for a long time, its chemical composition is influenced by many understudied factors, among which we only mention geographical position, climate, soil pH and so on [28]. 

For these reasons, studies on this plant are particularly important given its special therapeutic potential.

Additionally, some small peptides in the composition of this plant could be another key constituent to antitumor activity. 

Currently, there are several therapeutic approaches to cancer, including drugs, genetics and anticancer peptides. The results of studies regarding peptide anticancer therapy showed that the sequence of these small anticancer peptides can include several amino acids, such as: arginine, glycine, lysine and leucine, glutamic and aspartic, tyrosine, phenylalanine, proline and protonated histidine. Moreover, recent research has showed that arginine has a key role in the function of the immune system and antitumor activity [29,30,31].

Nevertheless, there are very few studies that have investigated the amino acid composition of Artemisia genus [32].

Studies regarding wild Romanian Artemisia genus are few and targeted, especially on bioactive components of *Artemisia absinthium* [33,34]. Furthermore, regarding Romanian *Artemisia annua* wild plant, the present research only investigates the content of artemisinin and volatile oils [35,36,37].

Despite its high therapeutic potential, the chemical screening of the biologically active compounds of this medicinal plant from the spontaneous flora of Romania has not been performed yet.

On the other hand, it should be mentioned that several herbal supplements of *Artemisia annua* are marketed globally, which are recommended, according to the manufacturers, for malaria, arthritis and even cancer.

Recently, several cases of liver disease have been reported, especially following self-medication with herbal supplements to manage cancer or prevent malaria [38,39].

The development of highly efficient, selective, simple and inexpensive nano-carrier systems could be an effective method to avoid these risks while ensuring the controlled intake of phytoconstituents with biological activity [40,41].

Modern drug delivery systems based on magnetic nanoparticles could easily accomplish these requirements. Moreover, the latest developments regarding magnetite nanoparticles have demonstrated their benefits and recommend their use in drug delivery and other different biomedical applications (magnetic resonance imaging, tumor therapeutic hyperthermia, etc.) [25,42].

Magnetic/superparamagnetic nanoparticles could represent a more than interesting alternative due to their advantages: their capability for local delivery and the ability to act selectively. However, the possibility of an immune response is the main drawback of these nanoparticles. Therefore, the design of new drug delivery systems based on magnetic/superparamagnetic nanoparticles for use as early detection methods and in the diagnosis, prognosis and monitoring of the evolution of the cancer treatment is required given the social impact of these diseases [25,43,44,45,46,47,48,49].

Recent studies have shown that nano-carriers based on magnetic nanoparticles lead to a high drug tissue permeability and retention effect and thus enhance the beneficial therapeutic effects [45,46,47].

To our best knowledge, this study investigates the metabolite profile of *Artemisia annua* grown wild in Romania for the first time. Subsequently, a simple and inexpensive nano-carrier system that capitalizes both the therapeutic properties of *Artemisia annua* (whole plant) and magnetic Fe_3_O_4_ nanoparticles was developed.

## 2. Results and Discussion 

Extensive research in the field of plants and especially on those with high therapeutic potential has shown that they have a very complex composition of compounds with high biological activity that act synergistically in the body [26,27].

Additionally, a full description of a general metabolic profile for a specific herb is all the more difficult, as significant differences in secondary metabolites was reported among the same plants harvested from various geographic regions of the world. Studies in this area confirmed that the content of specific plant secondary metabolites is the result of several environmental stress factors (climate, soil and biological conditions) which directly influence plant growth, development and topography distribution [28,50,51,52].

The pharmacological properties of different plant secondary metabolites were extensively investigated. However, their therapeutic benefits have not been completely understood [11,21,22]. Plant metabolites with peptide structures are just an example of this [51].

Bioactive metabolite chemical screening of sweet wormwood (năfurica) was tentatively carried out via gas-chromatography coupled with mass spectroscopy (GC-MS) and electrospray ionization–quadrupole time-of-flight mass spectrometry (ESI-QTOF-MS) analysis. 

**Figure 1:**
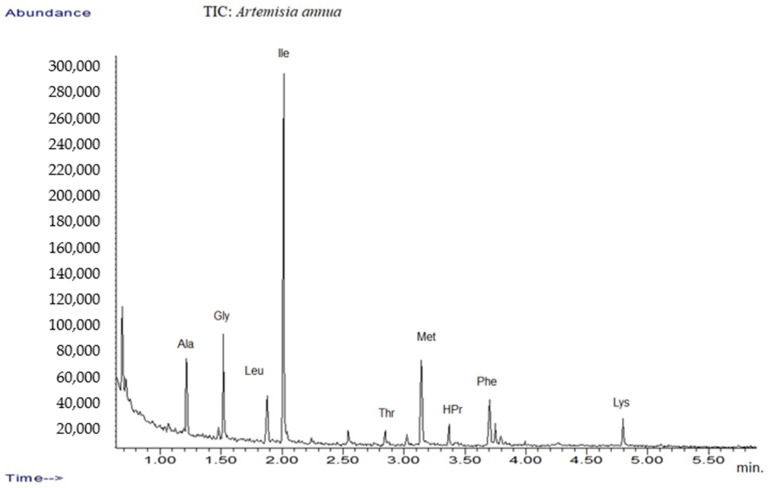
TIC chromatograms of GC-MS for *Artemisia annua*.

In addition, the amino acid profile was investigated using GC-MS techniques (Figure 1). The mass spectra of components identified were determined via comparison of their retention indices and mass spectra with those of NIST/EPA/NIH, the Mass Spectral Library 2.0 data base, as well as by reviewing the literature [32,53].

The results are listed in Table 1, which presents tentative amino acid identification via GC-MS corresponding to *Artemisia annua* sample [32,54,55].

### 2.1. Mass Spectrometry Analysis of Romanian Artemisia annua

**Figure 2:**
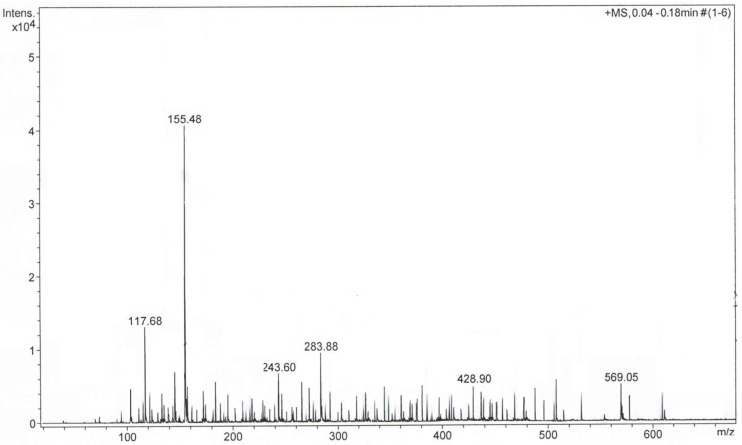
Positive ion mode MS-TOF of *Artemisia annua* sample.

The plant sample was diluted in methanol and analyzed using ESI-TOF mass spectroscopy (ESI-QTOF-MS). The plant extract sample analysis was carried out in the positive mode. The mass spectra (Figure 2) showed the presence of complex metabolite composition. A total of 103 compounds were detected and identified, which covered various chemical categories, including amino acids, sterols, terpenoids, flavonoids, coumarins, alcohols, aldehydes, glycosides, carbohydrates, fatty acids and so on, which confirmed the data reported in the literature [6,7,8,9,10,11,12,13,14,15,16,17,18,19,20,21,22,23,24,28,31,32,33,34,35,36,37,38,39,46,53,54,55,56,57,58,59,60]. Additionally, the presence of amino acids identified through GC-MS was confirmed via ESI-QTOF-MS analysis. However, only a few studies reported the amino acid profile of Artemisia annua [32].

The identified metabolites are listed in Table 2 and classified according to their m/z ratio (mass-to-charge-ratio) (theoretical and measured), chemical name and formula and the related literature.

### 2.2. Screening and Classification of the Differential Metabolites

The 103 phytochemicals identified through mass spectroscopy were assigned to different chemical classes: terpenoids and sesquiterpenoids (27.2%), flavonoids (24.2%), amino acids (12.6%), hydrocarbons (6.8%), coumarins (4.85%), phenolic acids (2.9%), sterol and steroids (2.9%), fatty acids (2.9%), glycosides (1.9%), hydrocarbons (6.8%), organic acids and esters (3.8%), carbohydrates (0.97%) and miscellaneous (Table 3). Terpenoids and sesquiterpenoids, flavonoids and amino acids constitute the largest group of bioactive compounds from *Artemisia annua*. The distribution of identified metabolites in various chemical categories is listed in Table 3.

**Figure 3:**
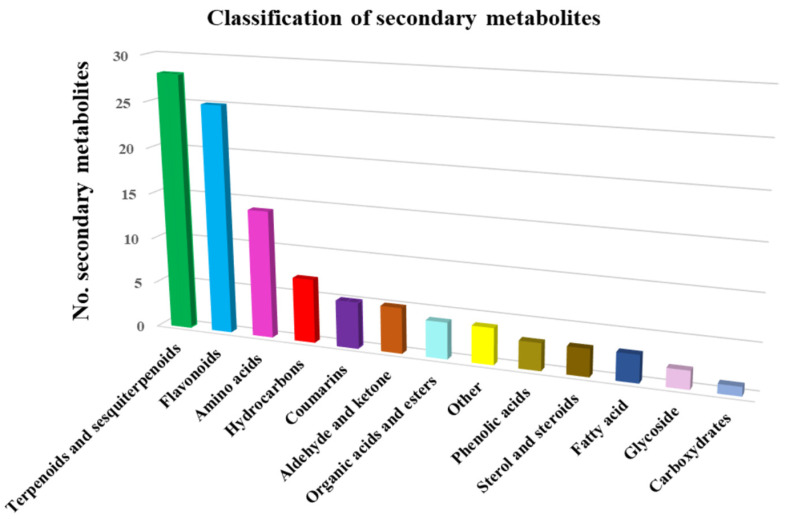
*Artemisia annua* metabolite classification.

On the basis of the data analysis reported in Table 3, the metabolites classification chart was obtained, represented in Figure 3.

Amino acids a total of 14 different amino acids were identified in the plant extract. Additionally, the essential amino acids (valine, leucine, methionine, hystidine and l-phenylalanine) represent 35% of them. The non-essential amino acids are present in a much larger proportion (65%), being representatives of: glycine, alanine, serine, arginine, threonine, acid aspartic, lysine and glutamic acid [31,32,33,73]. As was reported, amino acids exhibit antitumoral, antiproliferative and immunomodulant activity [31,32,33,73].

Terpenoids found in the *Artemisia annua* sample are the one of the major constituents of the total identified metabolites. Previous studies on the therapeutic effect of terpenoids have demonstrated their antimicrobial, antibacterial, antifungal, analgesic and anti-insect activity [74].

Sesquiterpenes, another important class of metabolites from *Artemisia annua*, were shown to have antitumoral, antiplasmodial, anti-inflammatory and anti-allergic properties. Sesquiterpenes lactones isolated from *Artemisia annua* are used in antimalaria drugs [75,76].

Coumarins are metabolites highly relevant to human health. Recent studies on coumarins isolated from plants have shown antioxidant, antimicrobial, antiviral, antifungal, and antiparasitic, anti-diabetic, analgesic, anti-neurodegenerative, and anti-inflammatory activity. Moreover, coumarins have been demonstrated to stimulate the immunologic response and are used in the therapy of different tumors: leukemia, renal and prostate tumors, melanoma and breast cancer [77,78].

Flavonoids were another major category of metabolites identified in the plant sample. A total of 25 different flavonoids were found in the *Artemisia annua* sample. These compounds exhibit antioxidant, antitumoral, anti-inflammatory, antimicrobial, anti-cholinesterase, neurodegenerative disease (Alzheimer) and atherosclerosis prevention effects [9,10,11,12,13,14,15,16,17,18,19,20,21,22,23,24,25,26,27,28,29,30,31,32,33,34,35,36,37,38,39,40,41,42,43,44,45,46,47,48,49,50,51,52,53,54,55,56,57,58,59,60,61,62,63,64,65,66,67,68,69,70,71,72,73,74,75,76,77,78,79,80,81].

Phenolic acids have shown anti-inflammatory, antioxidant, antimicrobial, neuroprotective, antidiabetic and anticancer effects [82,83].

Sterol and steroids from herbs act as antitumoral, anti-inflammatory, antioxidant antiatherosclerotic agents [84].

Fatty acids are involved in neuroprotection and cardiovascular protection mechanisms. Recent studies reported their beneficial role in autoimmune and neurodegenerative diseases, including Alzheimer disease (AD) [85].

Carbohydrates have shown anti-inflammatory, antioxidant, antiviral, antibacterial, antidiabetic, antitumoral, immunomodulatory and cardioprotective activity [86,87,88].

Glycosides from herbs showed antitumoral activity, mainly on leukemia and gastric cancer [89].

### 2.3. Nano-Carrier System Based on Magnetic Nanoparticles of Fe_3_O_4_

The development of an efficient and selective drug nano-carrier system required an optimal ratio between the herb and magnetic nanoparticles in order to provide the highest biological activity and functionality (selectivity and vectorization). Recent studies regarding types of nano-drug systems have reported the specific bioactive phytochemicals that were loaded into the magnetic nanoparticles [90,91].

### 2.4. FT-IR Spectroscopy

**Figure 4:**
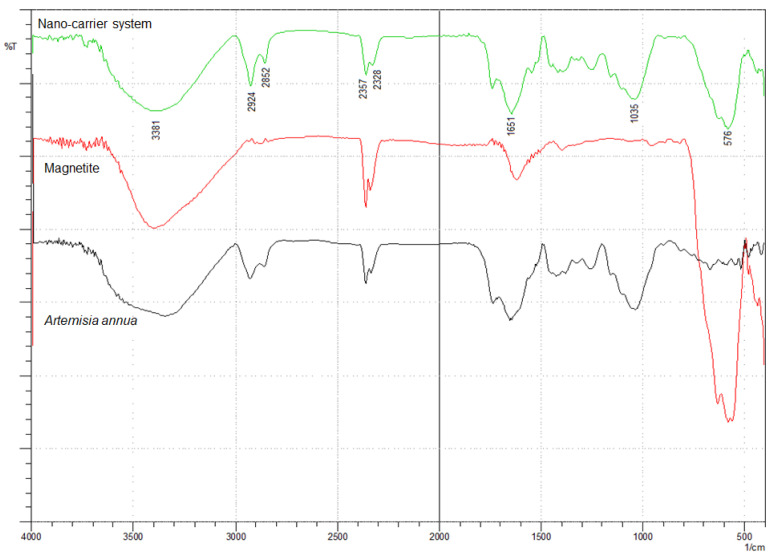
FT-IR spectra of *Artemisia annua*, magnetite nanoparticles and nano-carrier system.

The incorporation of herb phytochemicals into the pores of Fe_3_O_4_ nanoparticles was successfully achieved and was confirmed through FT-IR spectroscopy. Figure 4 presents the spectra of the herb, Fe_3_O_4_ nanoparticles and the nano-carrier system.

The FT-IR spectra of the herb display the characteristic absorption peaks of *Artemisia annua* (Figure 4). The characteristic group frequencies of different organic molecules detected in *Artemisia annua* can be attributed to flavonoids (1703, 1580, 1460, 630 and 575 cm^−1^), amino acids (1651, 1580, 1555 and 1545 cm^−1^), terpenoids (1740, 1651 and 810 cm^−1^), carbohydrates (3381, 1462, 1126 and 840 cm^−1^) and fatty acids (2925, 2852, 1250 and 720 cm^−1^) [47,92,93,94,95,96,97,98,99,100,101].

Withal, the band at 1737 cm^−1^ is the characteristic absorption peak of *Artemisia annua* assigned to δ-lactone group [47].

Two main broad metal–oxygen bands are seen in the IR spectra of Fe_3_O_4_ nanoparticles (Figure 4) in the range 400–600 cm^−1^. The highest vibration band at 576 cm^−1^ is assigned to the stretching vibrations of M_tetra_O bond in the tetrahedral voids, and the lowest band at 410 cm^−1^ (partially visible) corresponds to the stretching vibrations of the M_octa_O bond in the octahedral void peak [102,103,104].

The spectra of the nano-carrier system (Figure 4) display the characteristic peaks of the herb as well as the metal–oxygen vibration bands at 576 cm^−1^ and at 410 cm^−1^, which confirm the incorporation of the herb into the pores of Fe_3_O_4_ nanoparticles [47].

### 2.5. X-ray Diffraction Spectroscopy

**Figure 5:**
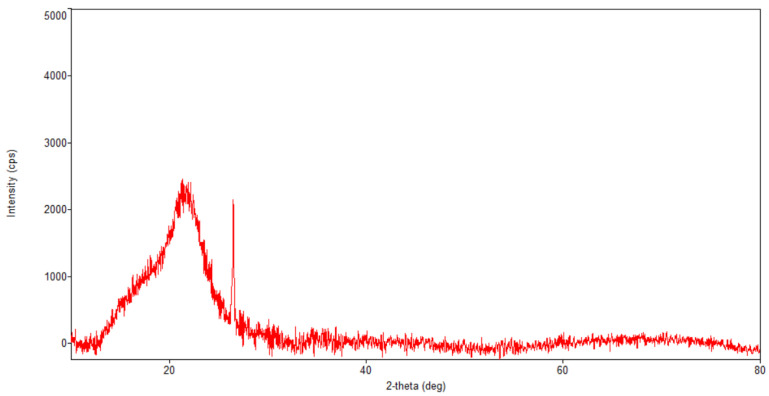
Powder XRD patterns of *Artemisia annua*.

**Figure 6:**
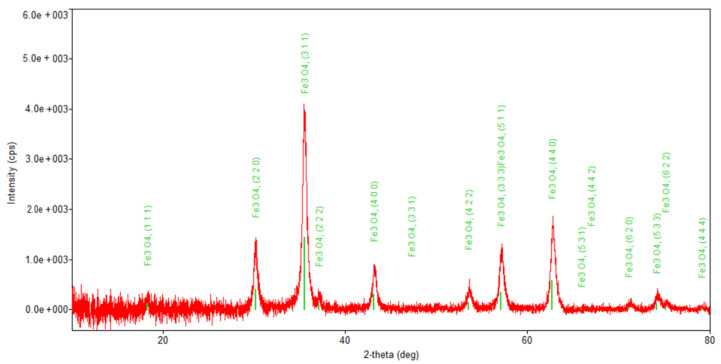
Powder XRD patterns of Fe_3_O_4_ nanoparticles.

**Figure 7:**
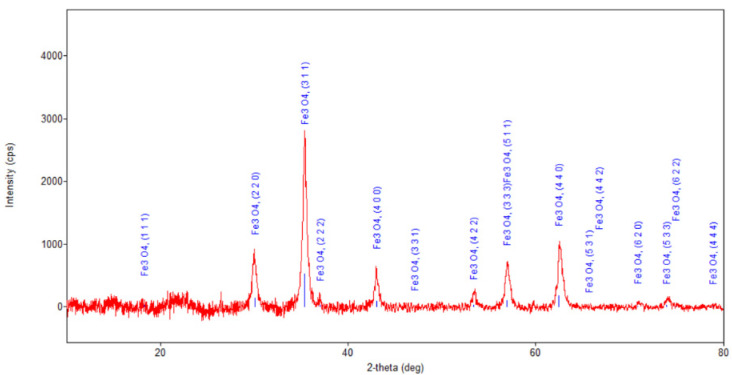
Powder XRD patterns of nano-carrier system.

**Figure 8:**
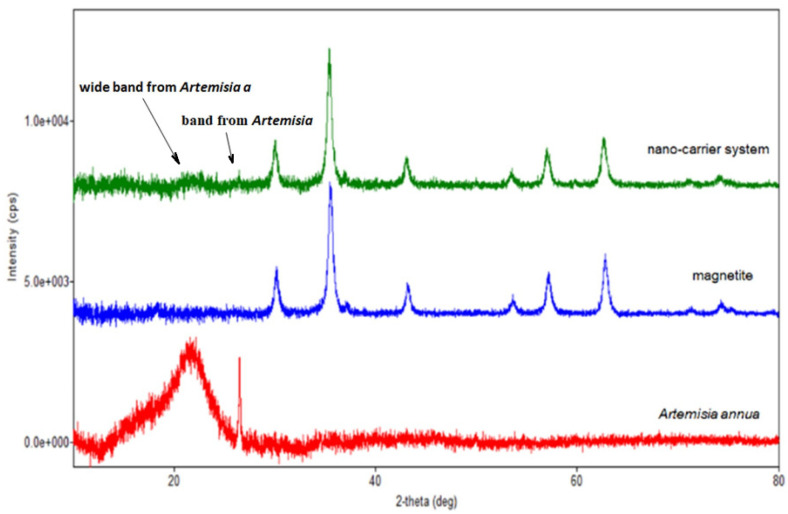
Overlap of the XRD patterns of herb, magnetite and nano-carrier system.

Figure 5, Figure 6, Figure 7 and Figure 8 present the XRD patterns of Fe_3_O_4_ nanoparticles, the herb and the nano-carrier system.

Regarding the diffraction pattern of the herb (Figure 5), in the range of 13–26°, a wide band that is characteristic of some amorphous phases can be observed. This wide band is also found attenuated in the diffraction pattern of the nano-carrier system (Figure 7 and Figure 8). Additionally, in the diffraction pattern of the Fe_3_O_4_ nanoparticles (Figure 6), only the peaks of the single crystalline spinel phase Fe_3_O_4_ (average crystallite size 10.9 nm) are present.

In the diffraction pattern of the mixture (Figure 7 and Figure 8), crystalline spinel phase Fe_3_O_4_ nanoparticles with an average crystallite size of 12.9 nm were identified. A peak at ~26.5° and a band between 13–26° were also present but much attenuated in the spectrum of *Artemisia annua*.

### 2.6. Scanning Electron Microscopy (SEM)

**Figure 9:**
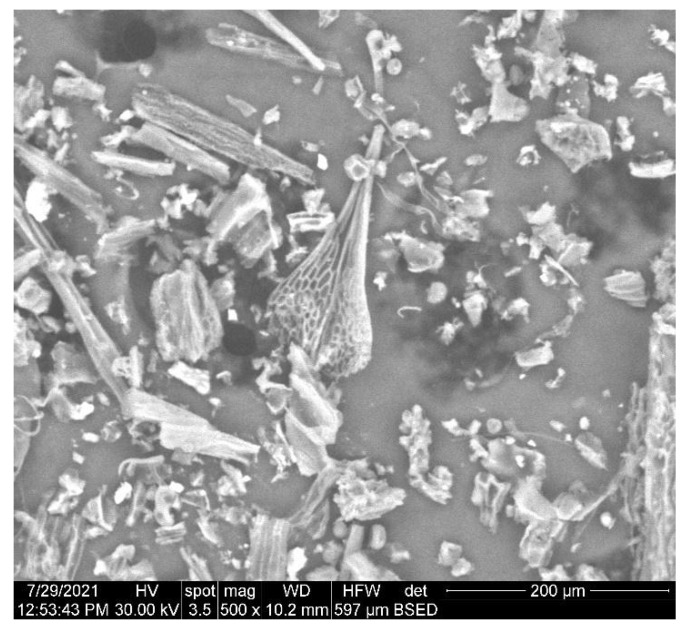
High-resolution SEM images of *Artemisia annua*.

**Figure 10:**
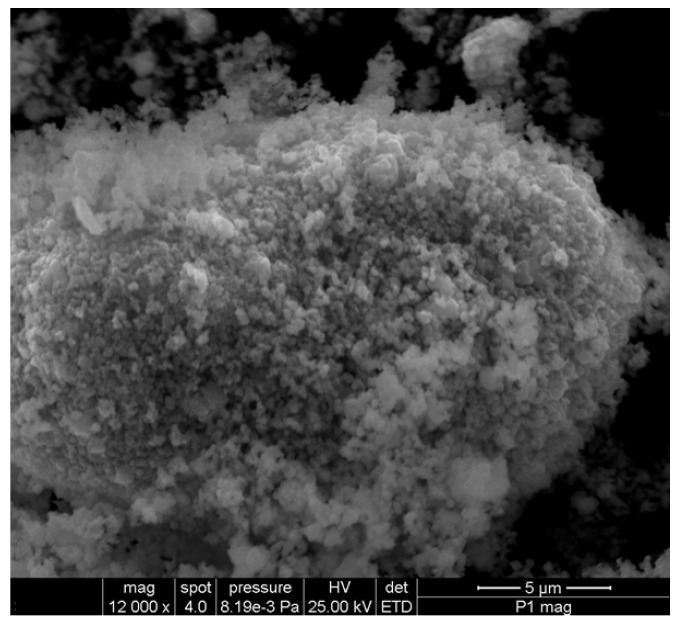
High-resolution SEM images of Fe_3_O_4_ nanoparticles.

**Figure 11:**
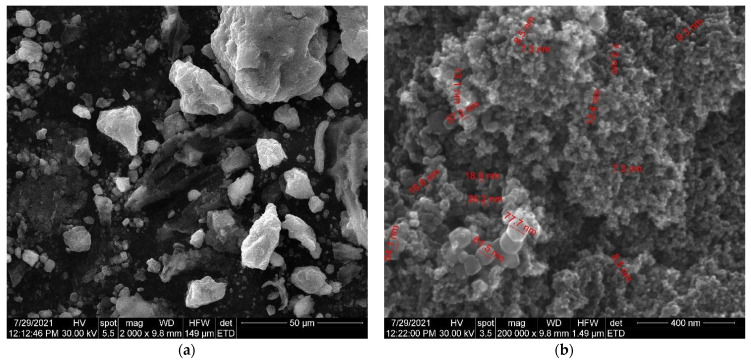
(**a**) SEM images of Fe_3_O_4_ nanoparticles loaded with herb at high magnification. (**b**) SEM images of Fe_3_O_4_ nanoparticles loaded with herb at low magnification.

The SEM micrographs of the herb, magnetic nanoparticles and the nano-carrier system are shown in Figure 9, Figure 10 and Figure 11.

As can be seen in the SEM image (Figure 9), the particles of *Artemisia anuua* shown are in the form of micron-sized fibers and irregular shape particles. The incorporation of herb phytochemicals into the pores of Fe_3_O_4_ nanoparticles was also confirmed via the scanning electron microscopy (SEM) images of Fe_3_O_4_ nanoparticles (Figure 10) and Fe_3_O_4_ loaded with the herb (Figure 11). The SEM image of Fe_3_O_4_ nanoparticles loaded with the herb (Figure 11b) shows a powder that consists of agglomerations of round nanoparticles with dimensions between 5 and 30 nm, as well as irregular shapes with dimensions greater than 60 nm. In the SEM image at high magnification (Figure 11a), irregular shape particles in Fe_3_O_4_ nanoparticles’ surface modification with herbs can be seen.

### 2.7. Magnetic Properties

**Figure 12:**
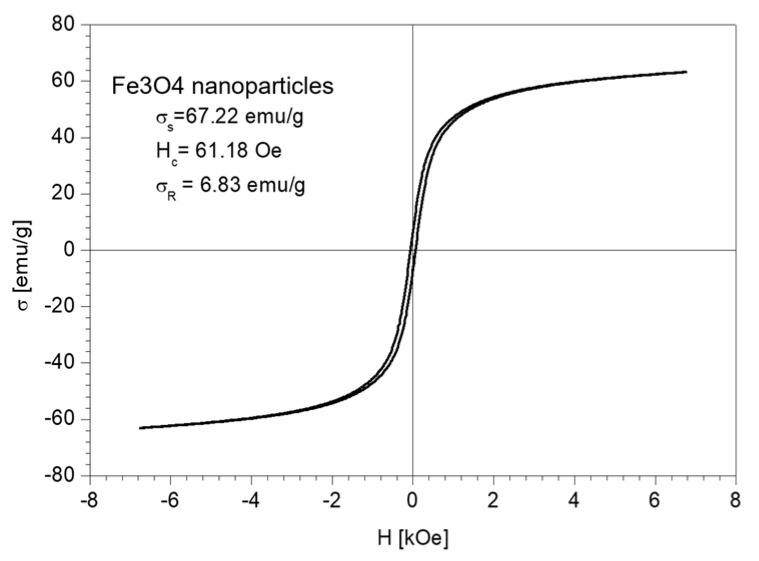
The hysteresis loop of Fe_3_O_4_ nanoparticles sample.

**Figure 13:**
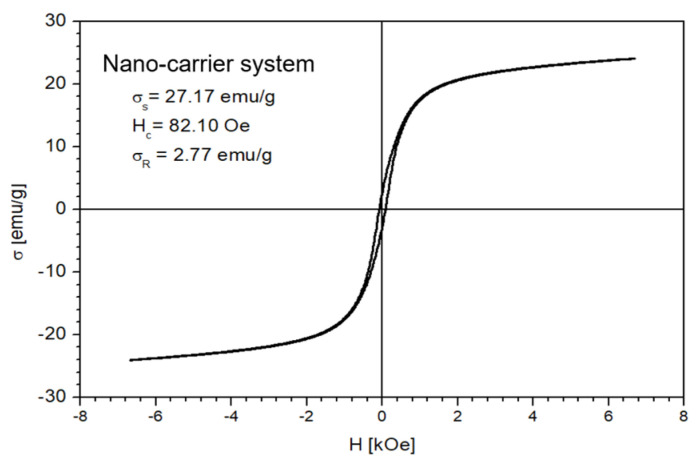
The hysteresis loop of nano-carrier system.

The magnetic properties of the Fe_3_O_4_ nanoparticles and the nano-carrier system were investigated at a low-frequency driving field (50 Hz) by means of an induction hysteresis graph [105]. It was found that both samples exhibit ferromagnetic behavior with narrow hysteresis loops (see Figure 12 and Figure 13).

From the measured hysteresis loops, the saturation magnetization (σ_S_), the coercive field (*H_c_*) and the remanent magnetization (σ_R_) were determined. The results are presented in Table 4.

**Figure 14:**
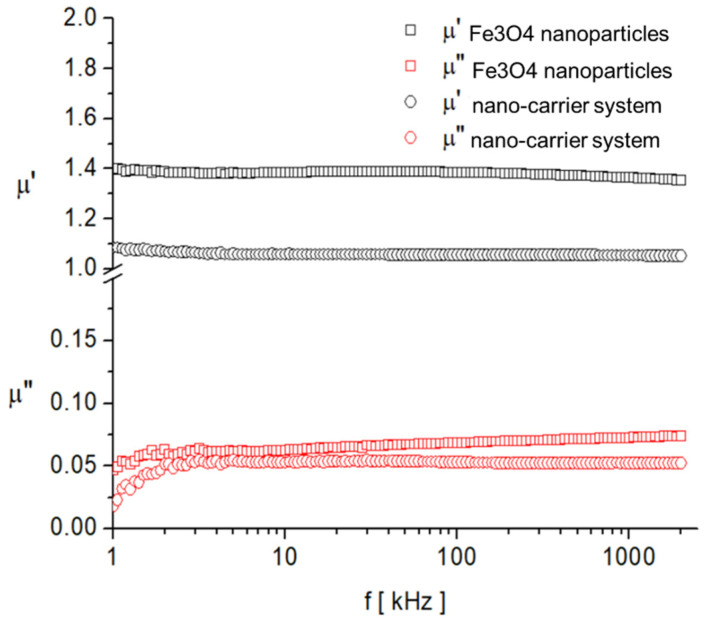
Frequency dependence of the real, $\mu^{\prime }$, and imaginary, $\mu^{\prime \prime }$, components of the complex magnetic permeability of samples.

As expected, the saturation magnetization of the sample Fe_3_O_4_ nanoparticles is larger than that of nano-carrier system. Both samples have small values regarding the remnant ratio σ_R_/σ_S_ (in order of 0.1), which is an indication of the ease with which the magnetization reorients to the nearest easy axis magnetization direction after the removal of the magnetic field. The frequency dependence on the complex magnetic permeability of the samples (Equation (1)) over the frequency range of 1 kHz to 2 MHz was measured at room temperature, and the obtained results are presented in Figure 14 [106].

(1) $$\mu \left(f\right)=\mu^{\prime }\left(f\right)-i\mu^{\prime \prime }\left(f\right)$$

where $\mu^{\prime }\left(f\right)$ is the real part;$\mu^{\prime \prime }\left(f\right)$ is the imaginary part.

In the investigated frequency range, there are no magnetic relaxation peaks that provide clues about the characteristic magnetization processes. However, given the small sizes of the particles and also the low value of the imaginary component of complex magnetic permeability, it can be assumed that the dominant magnetization mechanism is the Neel process, correlated with the rotation of the magnetic moment inside the particles by overcoming the magneto-crystalline anisotropy barrier [107,108].

The obtained results indicate that the nano-carrier system, wherein the selected ratio of plant:magnetic nanoparticles is 3:1, exhibits magnetic properties.

## 3. Materials and Methods

All used reagents were GC grade. Methanol and chloroform were purchased from VWR (Wien, Austria). The Fe_3_O_4_ nanoparticles (nanoparticle size: 23 nm) were provided by the National Research and Development Institute for Non-Ferrous and Rare Metals, Pantelimon, Romania. The plant samples (whole plant) were collected in August 2020 from the area of Timis, Romania and were taxonomically authenticated at Victor Babes University of Medicine and Pharmacy, Timisoara, Romania. The plant samples were rapidly frozen in liquid nitrogen (−194 °C), ground and sieved to obtain a particle size lower than 0.5 mm, and they were kept at −80 °C to avoid enzymatic conversion or metabolite degradation. For each analysis, 1.8 g of dried sample was subject to sonication extraction in 25 mL of solvent (methanol/chloroform = 1:1) for 25 min at 40 °C with a frequency of 50 kHz. The solution was concentrated using a rotavapor and the residue was dissolved in MeOH. The extract was centrifuged, and the supernatant was filtered through a 0.2 μm syringe filter and stored at –18 °C until analysis.

### 3.1. Nano-Carrier System Preparation

For each analysis, 1.5 g of sample was prepared from dried herb (whole plant, ground and sieved to obtain a particle size lower than 0.5 mm), and Fe_3_O_4_ nanoparticles were added (herb/Fe_3_O_4_ nanoparticles = 3:1). The obtained mixture was subjected to micronization at room temperature for 5 min.

### 3.2. GC-MS Analysis

Gas chromatography was carried out using ClarusSQ8 GC/MS (Perkin Elmer) apparatus with a ZB-AAA GC column (10 m × 0.25 mm) (Phenomenex, Torrance, CA, USA); carrier gas, He; flow rate, 1 mL/min, following 3.3. GC-MS Separation Conditions (the standard conditions provided with the EZ: faast GC-MS free amino acids kit).

The oven temperature was 80 °C (held for 9 min) to 220 °C (held for 5 min) at 320 °C/min (held for 10 min); the equilibration time was 1 min. The injection parameters were: split 1:5; 250 °C; 2.5 µL. The carrier gas was helium; 1.1 mL/min; 110 °C. The inlet pressure was 5.9 kPa/min; the detector used was: MS; mode: Scan Transfer Line; temperature: 250 °C; analyzer type: MS; electron energy: 70 eV.

### 3.3. Mass Spectrometry

MS experiments were conducted using EIS-QTOF-MS from Bruker Daltonics, Bremen, Germany. All mass spectra were acquired in the positive ion mode within a mass range of (100–3000) *m*/*z*, with a scan speed of 2.1 scans/s. The source block temperature was kept at 80 °C. The reference provided a spectrum in positive ion mode with fair ionic coverage of the *m*/*z* range scanned in full-scan MS. The resulting spectrum was a sum of scans over the total ion current (TIC) acquired at 25–85 eV collision energy to provide the full set of diagnostic fragment ions.

### 3.4. Identification of Metabolites

The total ion current (TIC) and selected ion monitoring (SIM) values were compared with those from Phenomenex-EZ: faast amino acid analysis user guide and the results are presented in Table 1. The metabolites were identified via comparison of their mass spectra with those of the standard library NIST/NBS-3 (National Institute of Standards and Technology/National Bureau of Standards) spectral database, and the identified phytoconstituents are presented in Table 2.

### 3.5. FT-IR Spectroscopy

The FT-IR spectrum of the sample was recorded via KBr pellet using a Shimadzu Prestige-21 spectrometer in the range 400–4000 cm^−1^, with a resolution of 4 cm^−1^.

### 3.6. XRD Spectroscopy

The phase composition of the sample was determined via powder X-ray diffractometry (XRD) using monochromatic CuKα radiation (λ = 1.5406Å) on a Rigaku Ultima IV diffractometer equipped with a D/teX Ultra detector and operating at 40 kV and 40 mA. The analysis was performed in the 2θ range of 10–80°, with a scan speed of 5 °/min and a step size of 0.01° 2θ. The average crystallite size was calculated using the whole pattern profile fitting method (WPPF). The XRD patterns were compared with those from the ICDD Powder Diffraction Database (ICDD file 04-015-9120).

### 3.7. Scanning Electronic Microscopy (SEM)

The SEM analyses were performed using an SEM-EDS system (QUANTA INSPECT F50) equipped with a field emission gun (FEG), 1.2 nm resolution and an energy dispersive X-ray spectrometer (EDS) with an MnK resolution of 133 eV.

### 3.8. Magnetization Experiments

The frequency dependence of the Fe_3_O_4_ nanoparticles and nano-carrier system was measured using an Agilent LCR-meter (E-4980A type) at room temperature over the frequency range (1 kHz to 2 MHz) and various values of polarizing field. The duration of the measurement into a constant magnetic field over the entire frequency range was about 40 s.

Complex magnetic susceptibility measurements were made using the short-circuited coaxial transmission line technique at different values of the polarizing field, *H*, over the range 0–170 kA/m and at the frequency range (100 MHz–6 GHz). The static magnetization measurements for the Fe_3_O_4_ nanoparticle sample and the nano-carrier system were performed using a ballistic galvanometer.

## 4. Conclusions

In the current study, the complete metabolite profiling of *A. annua* growing wild in Romania was accomplished. A total of 14 amino acids were identified for the for the first time in plant samples. The biological activities were discussed for each metabolite category. Furthermore, a simple and economical nano-carrier system was developed. A ratio of herb:magnetic Fe_3_O_4_ nanoparticles was used, which allowed for the synergic effect of * A. annua* bioactive compounds and its inorganic component properties to be taken advantage of. The morpho-structural characterization of the nano-carrier system was performed. In addition, the magnetic properties of the nano-carrier were evaluated. Further studies are necessary to investigate the biological properties and the bioavailability of the new nano-carrier system.

## Figures and Tables

**Table 1:** Main compounds identified by GC-MS analysis of plant extract.

Proposed Structure	Abbreviation	SIM (Selected Ion Monitoring)
Alanine	Ala	130, 70
Leucine	Leu	172, 86
Glycine	Gly	116, 74
Isoleucine	Ile	172, 130
Methionine	Met	203, 277
Phenylalanine	Phe	206, 190
Lysine	Lys	170, 128
Threonine	Thr	160, 101
4-Hydroxyproline	HYP	172, 86

**Table 2:** Phytoconstituents identified in *Artemisia annua* sample through MS analysis.

Compound Name	m/z Detected	Theoretic m/z	Formula	Tentative Identification	References
**1**	76.09	76.07	C_2_H_5_NO_2_+	Glycine	[32,61]
**2**	90.11	90.097	C_3_H_7_O_2_+	Alanine	[32,61]
**3**	103.19	103.17	C_6_H_14_O+	Hexanol	[7,8,10,18,23,28,33,36,37,62]
**4**	106.12	106.09	C_3_H_7_NO_3_+	Serine	[32,61]
**5**	113.10	113.09	C_4_H_4_N_2_O_2_+	Uracil	[7,8,10,18,24,30,35,38,39,61]
**6**	118.13	118.14	C_5_H_11_NO_2_+	Valine	[32,61]
**7**	120.05	120.03	C_4_H_9_NO_3_+	Threonine	[32,61]
**8**	129.23	129.21	C_8_H_16_O+	Caprylaldehyde	[7,8,18,23,28,32,36,37,62]
**9**	132.14	132.13	C_5_H_9_NO_3_+	l-hydroxyproline	[32,61]
**10**	132.17	132.18	C_6_H_13_NO_2_+	Leucine	[32,61]
**11**	134.09	134.10	C_4_H_7_NO_4_+	Aspartic acid	[32,61]
**12**	135.23	135.22	C_10_H_14_+	p-Cymene	[63]
**13**	136.21	136.19	C_7_H_5_NS+	Benzothiazole	[7,8]
**14**	137.25	137.23	C_10_H_16_+	Limonene	[9,10,11,12,14,19,20,22,23,46,52,59,63,64,65]
**15**	139.15	139.12	C_7_H_6_O_3_+	Salicylic acid	[7,8,9,18,23,28,33,36,37,62]
**16**	147.16	147.14	C_9_H_6_O_2_+	Coumarin	[9,10,11,12,14,19,20,22,23,46,52,59,60,63,64]
**17**	147.20	147.19	C_6_H_14_N_2_O_2_+	Lysine	[32,61]
**18**	148.12	148.13	C_5_H_9_NO_4_+	Glutamic acid	[32,61]
**19**	149.21	149.205	C_10_H_12_O+	4-Isopropylbenzaldehyde	[7,8,18,23,28,33,36,37]
**20**	150.22	150.20	C_6_H_11_NO_2_S+	Methionine	[32,61]
**21**	151.19	151.22	C_10_H_14_O+	Cuminol	[7,8,18,23,28,33,36,37,62]
**22**	153.25	153.233	C_10_H_16_+	Artemisia ketone	[5,6,7,9,11,12,13,14,16,19,20,21,22,23,35,46,58,59,60,63,65]
**23**	155.23	155.21	C_12_H_10_+	Capillene	[7,8,18,23,28,33,36,37,62]
**24**	155.27	155.25	C_10_H_18_O+	Geraniol	[9,10,11,12,14,19,20,22,23,46,52,59,61,63,64]
**25**	156.17	156.16	C_6_H_9_N_3_O_2_+	Histidine	[32,61]
**26**	157.29	157.26	C_10_H_20_O+	Menthol	[9,10,11,12,14,19,20,22,23,46,52,59,63,64,66]
**27**	162.15	162.13	C_9_H_5_O_3_+	4-Hydroxycoumarin	[7,8,10,18,23,28,33,36,37,62]
**28**	165.19	165.16	C_9_H_8_O_3_+	4-Hydroxycinnamic acid	[7,8,10,18,23,28,33,36,37,62]
**29**	165.18	165.20	C_10_H_12_O_2_+	Eugenol	[9,10,11,12,14,19,20,22,23,46,52,59,63,64,66]
**30**	166.22	166.19	C_9_H_11_NO_2_+	l-phenylalanine	[32,61]
**31**	171.29	171.33	C_12_H_26_+	Dodecane	[7,8,18,23,28,33,36,37,62]
**32**	175.19	175.20	C_6_H_14_N_4_O_2_+	l-arginine	[32,61]
**33**	181.17	181.16	C_9_H_8_O_4_+	Caffeic acid	[7,8,18,23,28,33,36,37,62]
**34**	183.19	183.17	C_9_H_10_O_4_+	2,4-Dihydroxy-6 methoxyacetophenone	[7,8,18,23,28,33,35,37]
**35**	193.15	193.17	C_10_H_8_O_4_+	Scopoletin	[9,10,11,12,14,19,20,22,23,46,52,59,63,64,66]
**36**	197.21	197.20	C_10_H_12_O_4_+	Xanthoxylin	[7,8,18,23,28,33,36,37,62]
**37**	197.30	197.29	C_12_H_20_O_2_+	Artemisyl acetate	[5,6,7,9,11,12,13,14,16,19,20,21,22,35,46,58,59,60,63,65]
**38**	207.21	207.19	C_11_H_10_O_4_+	Scoparone	[9,10,11,12,14,19,20,22,23,46,52,58,63,64,66]
**39**	205.33	205.35	C_15_H_24_+	Germacrene D	[9,10,11,12,14,19,20,22,23,46,52,58,63,64,66]
**40**	207.39	207.37	C_15_H_26_+	Cadinene	[9,10,11,12,14,19,20,22,23,46,52,58,63,64,66]
**41**	220.23	220.24	C_10_H_13_N_5_O+	Zeatin	[7,8,18,23,28,33,36,37,62]
**42**	221.36	221.35	C_15_H_24_O+	Spathulenol	[9,10,11,12,14,19,20,22,23,46,52,58,63,64,66]
**43**	223.38	223.37	C_15_H_26_O+	Farnesol	[9,10,11,12,14,19,20,22,23,46,52,58,63,64,66]
**44**	229.35	229.37	C_14_H_28_O_2_+	Myristic acid	[7,8,18,23,28,33,36,37,62]
**45**	233.30	233.32	C_15_H_20_O_2_+	Alantolactone	[64]
**46**	235.29	235.33	C_15_H_22_O_2_+	Artemisinic acid	[5,6,7,9,11,12,13,14,16,19,20,21,22,35,46,58,59,60,63,65]
**47**	237.37	237.35	C_15_H_24_O_2_+	Corymbolone	[9,10,11,12,14,19,20,22,23,46,52,59,63,64,66]
**48**	246.33	246.32	C_15_H_19_NO_2_+	Rupestine	[9,10,11,12,14,19,20,22,23,46,52,59,63,64,66]
**49**	265.31	265.32	C_15_H_20_O_4_+	Abscisic acid	[5,6,7,9,11,12,13,14,16,19,20,21,22,35,46,58,59,60,63,65]
**50**	241.48	241.5	C_17_H_36_+	Heptadecane	[7,8,18,23,28,33,36,37,62]
**51**	247.29	247.30	C_15_H_18_O_3_+	α-Santonin	[64]
**52**	249.30	249.32	C_15_H_20_O_3_+	Arteannuin B	[5,6,7,9,11,12,13,14,16,19,20,21,22,35,46,58,59,60,63,65]
**53**	255.35	255.5	C_18_H_38_+	n-Octadecane	[7,8,18,23,28,33,36,37,62]
**54**	257.43	257.42	C_16_H_32_O_2_+	Palmitic acid	[7,8,18,23,28,33,36,37,62]
**55**	263.42	263.40	C_17_H_26_O_2_+	α-Bergamotol acetate	[7,8,18,23,28,33,36,37,62]
**56**	267.30	267.33	C_15_H_22_O_4_+	Germacranolide	[64]
**57**	271.39	271.40	C_15_H_10_O_5_+	Apigenin	[56,57,58,59,60,67,68,69,70,71]
**58**	273.27	273.25	C_15_H_12_O_5_+	Naringenin	[56,57,58,59,60,67,68,69,70,71]
**59**	281.34	281.32	C_15_H_20_O_5_+	Artemisitene	[5,6,7,9,11,12,13,14,16,19,20,21,22,35,46,58,59,60,63,65]
**60**	283.34	283.33	C_15_H_22_O_5_+	Artemisinine	[5,6,7,9,11,12,13,14,16,19,20,21,22,35,46,58,59,60,63,65]
**61**	285.27	285.26	C_16_H_12_O_5_+	Acacetin	[56,57,58,59,60,67,68,69,70,71]
**62**	287.26	287.24	C_15_H_10_O_6_+	Luteolin	[56,57,58,59,60,67,68,69,70,71]
**63**	289.27	289.25	C_15_H_12_O_6_+	Eriodyctiol	[56,57,58,59,60,67,68,69,70,71]
**64**	297.51	297.50	C_20_H_40_O+	Phytol	[9,10,11,12,14,19,20,22,23,46,52,59,63,64,66]
**65**	301.28	301.26	C_16_H_12_O_6_+	Rhamnocitrin	[56,57,58,59,60,67,68,69,70,71]
**66**	303.21	303.23	C_15_H_10_O_7_+	Quercetin	[56,57,58,59,60,68,69]
**67**	305.22	305.25	C_15_H_12_O_7_+	Taxifolin	[56,57,58,59,60,64,67,68,69,70,71]
**68**	315.31	315.29	C_17_H_14_O_6_+	Cirsimaritin	[56,57,58,59,60,64,67,68,69,70,71]
**69**	317.24	317.26	C_16_H_12_O_7_+	Rhamnetin	[56,57,58,59,60,67,68,69,70,71]
**70**	317.37	317.40	C_19_H_24_O_4_+	Capillartemisin B	[56,57,58,59,60,64]
**71**	319.20	319.23	C_15_H_10_O_8_+	Quercetagetin	[56,57,58,59,60,64,67,68,69,70,71]
**72**	324.57	324.60	C_22_H_44_O+	2-Docosanone	[7,8,18,23,28,33,36,37,62]
**73**	331.26	331.29	C_17_H_14_O_7_+	Rhamnazin	[56,57,58,59,60,64,67,68,69,70,71]
**74**	333.24	333.26	C_16_H_12_O_8_+	Laricitrin	[56,57,58,59,60,64,67,68,69,70,71]
**75**	339.67	339.70	C_24_H_50_+	n-Tetracosane	[7,8,18,23,28,33,36,37,62]
**76**	345.32	345.30	C_18_H_16_O_7_+	Eupatorine	[56,57,58,59,60,64,67,68,69,70,71]
**77**	347.27	347.30	C_17_H_14_O_8_+	Syringetin	[56,57,58,59,60,64,67,68,69,70,71]
**78**	353.68	353.70	C_25_H_52_+	n-Pentacosane	[7,8,18,23,28,33,36,37,62]
**79**	354.29	354.31	C_16_H_18_O_9_+	Scopoline	[9,10,11,12,14,19,20,22,23,46,52,59,63,64,66]
**80**	359.31	359.30	C_19_H_18_O_7_+	Retusin	[56,57,58,59,60,64,67,68,69,70,71]
**81**	361.33	361.30	C_18_H_16_O_8_+	Chrysosplenol D	[56,57,58,59,60,64,67,68,69,70,71]
**82**	367.71	367.7	C_26_H_54_+	n-Hexacosane	[7,8,18,23,28,33,36,37,62]
**83**	375.28	375.30	C_19_H_18_O_8_+	Chrysosplenetin	[56,57,58,59,60,64,67,68,69,70,71]
**84**	389.39	389.40	C_20_H_20_O_8_+	Artemitin	[9,16,17,21,23,56,57,58,59,60,65]
**85**	375.28	375.30	C_19_H_18_O_8_+	Casticin	[56,57,58,59,60,64,67,68,69,70,71]
**86**	377.41	377.40	C_20_H_24_O_7_+	Euparotin	[56,57,58,59,60]
**87**	411.68	411.70	C_30_H_50_+	Squalene	[9,10,11,12,14,19,20,22,23,46,52,59,63,64,66]
**88**	413.72	413.70	C_29_H_48_O+	Stigmasterol	[7,8,18,24,30,35,38,39]
**89**	415.67	415.70	C_29_H_50_O+	β-Sitosterol	[7,8,18,23,28,33,36,37,62]
**90**	425.69	425.70	C_30_H_48_O+	Taraxasterone	[9,10,11,12,14,19,20,22,23,46,52,59,63,64,66]
**91**	427.71	427.70	C_30_H_50_O+	Beta-amyrin	[9,10,11,12,14,19,20,22,23,46,52,59,63,64,66]
**92**	433.37	433.40	C_21_H_20_O_10_+	Apigenin 7-*O*-glucoside	[64]
**94**	443.52	443.50	C_25_H_30_O_7_+	Tomentin A	[9,10,11,12,14,19,20,22,23,46,52,59,63,64,66]
**95**	447.38	447.40	C_22_H_22_O_10_+	Kaempferide 3-rhamnoside	[57]
**96**	449.37	449.40	C_21_H_20_O_11_+	Cymaroside	[56,57,58,59,60,64,67,68,69,70,71]
**97**	457.71	457.70	C_30_H_48_O_3_+	Oleanic acid	[7,8,18,22,27,31,34,35,60]
**98**	465.38	465.40	C_21_H_20_O_12_+	Isoquercetin	[56,57,58,59,60,64,67,68,69,70,71]
**99**	495.43	495.40	C_22_H_22_O_13_+	Patuletin 3-glucoside	[64]
**100**	505.37	505.40	C_18_H_32_O_16_+	Sophorotriose	[64]
**101**	517.42	517.40	C_25_H_24_O_12_+	Cynarine	[7,8,18,22,27,31,34,35,60]
**102**	577.77	577.80	C_35_H_60_O_6_+	Daucosterol	[7,8,18,22,27,31,34,35,60]
**103**	611.51	611.50	C_27_H_30_O_16_+	Rutin	[56,57,58,59,60,64,67,68,69,70,71,72]

**Table 3:** Classification of metabolites from *Artemisia annua* sample on various chemical categories.

Chemical Class	Metabolite Name
Amino Acids	Glycine
	Alanine
	Serine
	Valine
	Threonine
	l-hydroxyproline
	Leucine
	Aspartic acid
	Lysine
	Glutamic acid
	Methionine
	Hystidine
	l-phenylalanine
	l-arginine
Terpenoids and Sesquiterpenoids	Artemisine
	Artemisinine
	Limonele
	p-Cymene
	Beta-amyrin
	Artesimic acid
	Eugenol
	Menthol
	Artemisia ketone
	Spathulenol
	Artemisyl acetate
	Artemisinic acid
	Phytol
	Rupestine
	α-Santonin
	Arteannuin B
	Farnesol
	Corymbolone
	Abscisic acid
	Alantolactone
	Artemisitene
	Geraniol
	Squalene
	Taraxasterone
	Beta-amyrin
	Germacranolide
	Germacrene D
	Cadinene
Coumarins	Scopoletin
	Tomentin A
	Coumarin
	Scopolin
	Scoparone
Flavonoids	Apigenin
	Chrysosplenetin
	Rhamnazin
	Luteolin
	Naringenin
	Capillartemisin B
	Rutin
	Quercetin
	Quercetagetin
	Acacetin
	Rhamnetin
	Eupatorin
	Syringetin
	Laricitrin
	Eriodictiol
	Casticin
	Chrysosplenol D
	Retusin
	Cynaroside
	Artemitin
	Taxifolin
	Isoquercetin
	Rhamnocitrin
	Kaempferide 3-rhamnoside
	Cirsimaritin
Phenolic Acids	4-Hydroxycoumarin
	4-Hydroxycinnamic acid
	Caffeic acid
Sterol and Steroids	β-Sitosterol
	Stigmasterol
	Daucosterol
Fatty Acid	Oleanic acid
	Palmitic acid
	Myristic acid
Hydrocarbons	n-Octadecane
	Heptadecane
	n-Tetracosane
	n-Hexacosane
	Dodecane
	n-Pentacosane
	Capillene
Glycoside	Patuletin 3-glucoside
	Apigenin 7-*O*-glucoside
Carbohydrates	Sophorotriose
Aldehyde and Ketone	Caprylaldehyde
	4-Isopropylbenzaldehyde
	Hexanol
	2-Docosanone
	2,4-Dihydroxy-6-methoxyacetophenone
Organic Acids and Esters	Salicylic acid
	α-Bergamotol acetate
	Xanthoxylin
	Cynarine
Other	Uracil
	Cuminol
	Benzothiazole
	Zeatin

**Table 4:** Magnetic properties of Fe_3_O_4_ nanoparticles and nano-carrier system.

Sample	σ_S_ (emu/g)	H_C_ (Oe)	σ_R_ (emu/g)
Fe_3_O_4_ nanoparticles	67.22	61.18	6.83
Nano-carrier system	27.17	82.10	2.77

## Data Availability

All data are contained within the article.
